# Supplementary material for: An integrated text mining framework for metabolic interaction network reconstruction
Source: PeerJ. 2016 Mar 21;4:e1811. doi: 10.7717/peerj.1811 (PMC4806637; doi:10.7717/peerj.1811)
Supplement: File S1 [file peerj-04-1811-s001.pdf]

ADDITIONAL FILE 1: Pseudocode for conversion from event interaction graph to metabolic interaction graph (Figure 3B-b1 and 3B-b2) used in MINR module development.

```
INPUT: Pairs metabolic event
OUTPUT: The metabolic interaction network

metabolic_events = Module1.extract_metabolic_events(texts)
# Initializing the metabolic interaction network

# Querying each event arguments.
foreach metabolic_event_ in metabolic_event_pairs_:

    if type_of(metabolic_event) is "Positive_regulation":
        skip() # Skipping regulation node.

    #Initializing node for the metabolic interaction reconstruction.

    substrates = []     #List of substrate nodes
    products   = []     #List of product nodes
    enzymes =    []     #List of enzymes nodes

    theme_nodes = get_neighbor_by_edge_type(eventnode, "Theme")
    product_nodes = get_neighbor_by_edge_type(eventnode, "Product")

    cause_nodes = get_neighbor_by_edge_type(eventnode, "Cause")
    positive_regulation_node = get_neighbor_by_node_type(eventnode, "positive_regulation")

    #Adding a proxy node for if enzyme was not specified.
    if enzymes is empty:
        proxy_node = create_proxy_node()
    #Adding enzyme node
    if exists(cause_nodes):
        extends(enzymes, cause_nodes)
    if exists(positive_regulation_node):
        cause_nodes = get_neighbor_by_edge_type(positive_regulation_node, "Cause")
        extends(enzymes, cause_nodes)

    #Adding the substrate and the product nodes.
    if type_of(eventnode) is metabolic_conversion:
        extends(substrates, theme_nodes)
    if type_of(eventnode) is metabolic_consummption:
        extends(substrates, theme_nodes)
    if type_of(eventnode) is metabolic_production:
        extends(products, theme_nodes)

    #Building a metabolic reaction (Substrate -> Enzyme -> Product).
    foreach substrate in substrates:
        connect_from_to(substrate, enzyme)
    foreach product in products:
        connect_from_to(enzyme, product)
    #Add these reaction into the metabolic_interaction_network.

    append(metabolic_interaction_network, substrate, enzyme, product)

return metabolic_interaction_network
```
