# Supplementary material for: An integrated text mining framework for metabolic interaction network reconstruction
Source: PeerJ. 2016 Mar 21;4:e1811. doi: 10.7717/peerj.1811 (PMC4806637; doi:10.7717/peerj.1811)
Supplement: File S2 [file peerj-04-1811-s002.pdf]

<u>ADDITIONAL FILE 2</u>: List of manually metabolic interaction used for MINR module evaluation on test corpus.

Additional Table 2.1 - List of metabolic interaction in Superpathway of leucine, valine, and isoleucine biosynthesis

PMID-13405870		
	L-serine	L-serine deaminase
	L-threonine	L-threonine deaminase
PMID-13727223		
	a,b-dihydroxy-isovalerate	dihydroxy acid hydrase
	dihydroxy acid hydrase	a-ketoisovalerate
	a,b-dihydroxy b-methylvalerate	dihydroxy acid hydrase
	dihydroxy acid hydrase	a-keto b-methylvalerate
PMID-16326011		
	acetohydroxy acid synthase	acetolactate
	acetolactate	acetohydroxy acid synthase
	thiamine diphosphate	acetohydroxy acid synthase
	pyruvate	acetohydroxy acid synthase
	acetohydroxy acid synthase	ThDP-bound active intermediate
	ThDP-bound active intermediate	acetohydroxy acid synthase
	Ketobutyrate	acetohydroxy acid synthase
	acetohydroxy acid synthase	acetohydroxy butyrate
PMID-1646790		
	2-ketoisocaproate	ilvE
	2-ketoisocaproate	tyrB
	ilvE	leucine
	tyrB	leucine
	2-ketoisocaproate	TrB
	2-ketoisocaproate	TrD
	TrB	leucine
	TrD	leucine
PMID-16743051		
	threonine	l-threonine hydrolyase
	l-threonine hydrolyase	2-oxybyutyrate
	l-threonine hydrolyase	NH3
	l-threonine	l-threonine 3-dehydrogenase
	l-threonine 3-dehydrogenase	2-amino 3-oxybutyrate
	2-amino 3-oxybutyrate	coa-ligase
	CoA-ligase	acytyl CoA
	CoA-ligase	glycine
PMID-2007556		
None		
PMID-2653423		

	acetolactate	ketolacid reductoisomerase
	NADPH	ketolacid reductoisomerase
	ketolacid reductoisomerase	3-hydroxy-3methyl-2-oxobutyrate
	reductoisomerase	pantoate
	ketopantoate	reductoisomerase
PMID-2675968		
	acetolactate	acetohydroxy acid synthase
	acetohydroxy acid synthase	pyruvate
	acetohydroxybutyrate	acetohydroxy acid synthase
	acetohydroxy acid synthase	2-ketobutyrate
PMID-355220		
	threonine	threonine dehydrogenase
	threonine	threonine deaminase
	alpha-amino-beta-ketobutyrate	threonine deaminase
	threonine dehydrogenase	alpha-amino-beta-ketobutyrate
	glycine	threonine dehydrogenase
	threonine deaminase	glycine
	threonine	threonine dehydrogenase
	threonine deaminase	isoleucine
	threonine	threonine deaminase
	threonine deaminase	aminoacetone
	threonine dehydrogenase	aminoacetone
	threonine	threonine dehydrogenase
	threonine dehydrogenase	alpha-amino-beta-ketobutyrate
	threonine	threonine aldolase
	threonine aldolase	acetaldehyde
	threonine aldolase	glycine
	threonine	threonine deaminase
	threonine deaminase	alpha-ketobutyrate
	threonine deaminase	NH4+
PMID-370104		
	pyruvate	acetohydroxy acid synthase
	acetohydroxy acid synthase	acetolactate
	alpha-ketobutyrate	acetohydroxy acid synthase
	acetohydroxy acid synthase	acetohydroxybutyrate
PMID-378964		
	alpha-ketoglutarate	transaminase B
	transaminase B	l-isoleucine
	transaminase B	l-valine
	transaminase B	l-phynylalanine
	transaminase B	l-tyrosine
	aspartate	aspartate transaminase
	glutamate	aspartate transaminase
	valine	transaminase C
	alanine	transaminase C

	aminobutyrate	transaminase C
	glutamate	transaminase B
	leucine	transaminase B
	isoleucine	transaminase B
	valine	transaminase B
	phynylalanine	transaminase B
	methionine	transaminase B
PMID-4562389		
	2-acetolactate	alpha-acetohydroxy acid isomeroreductase
	alpha acetohydroxybutyrate	alpha-acetohydroxy acid isomeroreductase
PMID-4573981		
	L-threonine	L-threonine deaminase
PMID-5076220		
	threonine	threonine aldolase
	threonine	threonine dehydrogenase
	threonine	threonine dehydratase
	threonine dehydratase	2-oxobutyrate
	threonine dehydratase	propionate
	threonine	threonine dehydrogenase
	threonine	2-amino-3 oxobutyrate-CoA ligase
PMID-6154938		
	None	
PMID-6171647		
	None	
PMID-6195343		
	None	
PMID-8325851		
	None	

Additional Table 2.2 - List of metabolic interaction in Superpathway of pyridoxal 5'-phosphate biosynthesis and salvage

PMID-1268115		
	PNP synthase	PNP
	4-phosphohydroxyl-l-threonine	PdxA
	PdxA	1-amino-acetone-3 phosphate
	DXP	PdxJ
	1-amino-acetone-3 phosphate	PdxJ
	PdxJ	PNP
	PdxJ	Pi
	PNP	PdxH
	PdxH	PLP
PMID-12896974		
	HTP	PdxA
	PdxA	3-amino-2-oxopropyl phosphate
	3-amino-2-oxopropyl phosphate	PdxJ
	deoxyxylulose 5-phosphate	PdxJ
	PdxJ	PNP
	PdxJ	Pi
	L-threonine	threonine dehydrogenase
	threonine dehydrogenase	L-2-amino-3-keto-butyrate
	L-2-amino-3-keto-butyrate	2-amino-3-ketobutyrate Coa ligase
	2-amino-3-ketobutyrate Coa ligase	glycine
	2-amino-3-ketobutyrate Coa ligase	acetyl Coa
	oxalosuccinate	isocitrate dehydrogenase
	PNP	PNP oxidase
	PNP phosphate oxidase	PLP
	PMP	PNP oxidase
	PNP oxidase	PLP
PMID-15242009		
	None	
PMID-1537800		
	4-hydroxythreonine	PdxA
	D-1-deoxyxylulose	PdxA
	4-hydroxythreonine	pdxJ
	D-1-deoxyxylulose	pdxJ
	PdxA	PN
	PdxJ	PN
	PN	PN kinase
	PN kinase	PNP
	PNP	pyridoxine phosphate oxidase

	pyridoxine phosphate oxidase	PLP
	PN	transaminase
	transaminase	PMP
	PM	PN/PL/PM kinase
	PN/PL/PM kinase	PMP
	PMP	PNP/PMP oxidase
	PM	PN kinase
	PN kinase	PMP
	PMP	transaminase
	transaminase	PLP
PMID-17822383		
	deoxyxylulose 5-phosphate	PdxA
	4-phosphhydroxy-l-threoine	PdxA
	deoxyxylulose 5-phosphate	PdxJ
	4-phosphhydroxy-l-threoine	PdxJ
	PdxA	PLP
	PdxJ	PLP
PMID-323431		
	None	
PMID-8764513		
	PL	PN kinase
	PM	PN kinase
	PN kinase	PLP
	PN kinase	PMP
	PN	PN kinase
	PN kinase	PNP
	PNP	PNP/PMP oxidase
	PNP/PMP oxidase	PLP
PMID-953780		
	PNP	PdxH oxidase
	PLP	PdxH oxidase
	PL	PL kinase
	PL kinase	PLP
	PN	PN kinase
	PN kinase	PNP
	PM	PM kinase
	PM kinase	PMP
	PNP	PdxH oxidase
	PMP	PdxH oxidase
PMID-9696782		
	4-phosphohydroxy-L-threonine	PdxA
	PdxA	PNP
	1-deoxy-d-xylulose-5-phosphate	PdxJ
	PdxJ	PNP
	PNP	pdxH

	pdxH	PLP
	PLP	transminase
	transminase	PMP
	PMP	transminase
	PMP	PdxH oxidase
	transminase	PLP
	PdxH oxidase	PLP
	d-erythrose-4-phosphate	E4P dehydrogenase
	E4P dehydrogenase	4PE
	4PE	PdxB dehydrogenase
	4PE	PdxF
	PdxB dehydrogenase	4PHT
	PdxF	4PHT
	4PE	PdxB
