# Supplementary material for: An integrated text mining framework for metabolic interaction network reconstruction
Source: PeerJ. 2016 Mar 21;4:e1811. doi: 10.7717/peerj.1811 (PMC4806637; doi:10.7717/peerj.1811)
Supplement: File S3 [file peerj-04-1811-s003.pdf]

ADDITIONAL FILE 3: Top ten list of event words identified in the ME corpus

Additional Table 3.1 – Event word counts in the ME corpus

Event words	Event words count (%)
Catalyzes (V)	47 (9.75%)
Biosynthesis (N)	35 (7.26%)
Synthesis (N)	30 (6.22%)
Formation (N)	23 (4.77%)
Hydrolysis (N)	19 (3.94%)
Conversion (N)	16 (3.32%)
Utilization (N)	14 (2.90%)
Catalyzed (V)	13 (2.70%)
Catalyze (V)	12 (2.49%)
Metabolism (N)	12 (2.49%)
Total	221 (45.84%)

For each event word, its type is given in ((N)ominalised form and (V)erb form) together with an indication of the number of event words and the percentage of event words.
