# Supplementary material for: An integrated text mining framework for metabolic interaction network reconstruction
Source: PeerJ. 2016 Mar 21;4:e1811. doi: 10.7717/peerj.1811 (PMC4806637; doi:10.7717/peerj.1811)
Supplement: File S4 [file peerj-04-1811-s004.pdf]

<u>ADDITIONAL FILE 4:</u> Example results of metabolic interaction list achieved between manual curation and MINR module.

Additional Text and Table 4.1- Example result of metabolic interaction from PMID: 13405870

Sentences selected by a manual curator

S1: The identity of L-serine and L-threonine deaminase in *Escherichia coli* with a single enzyme protein was suggested by Wood and Gunsalus (1949), who were unable to effect any separation of the two activities following extensive purification.

S2: Evidence is presented here for recognition in *E. coli* extracts of two distinctly different enzymes catalyzing the deamination of L-threonine.

Result achieved from Module 2 within integrated TM framework

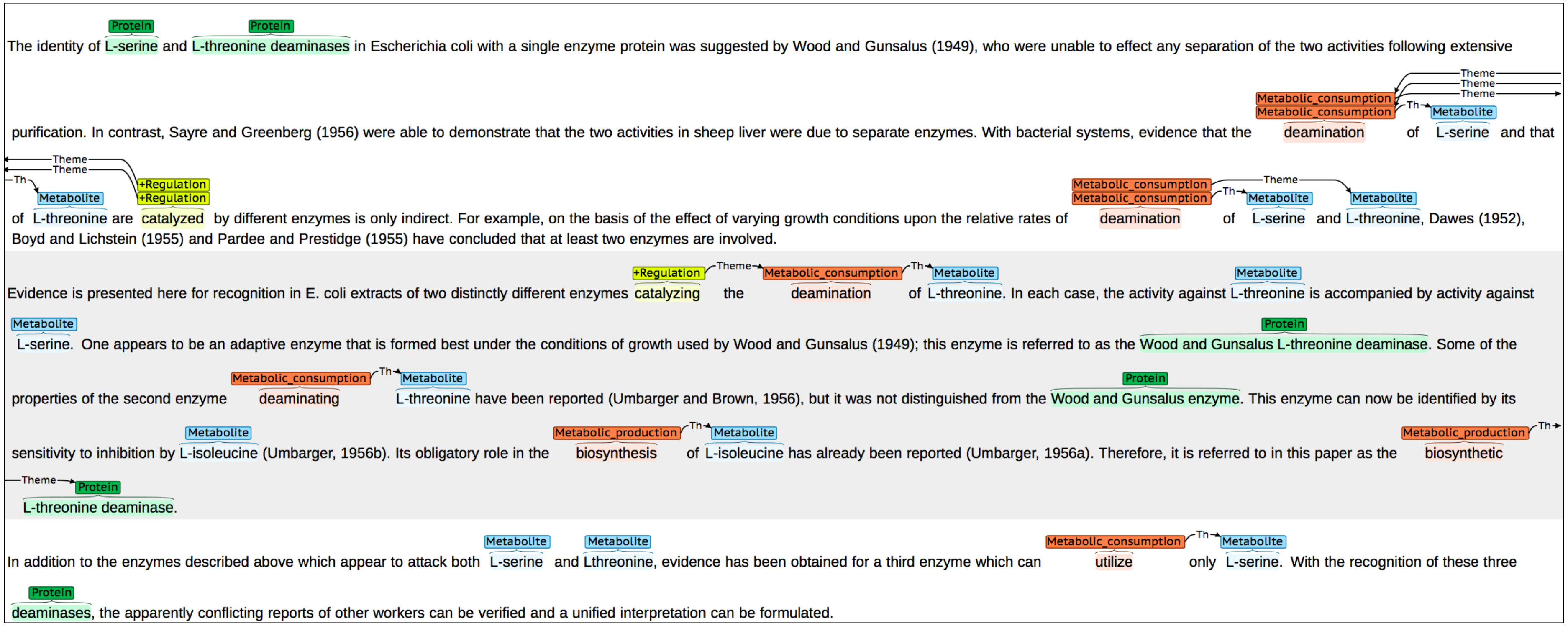

**Manual curation**		**Module 2**
L-serine	L-serine deaminase	Not found
L-threonine	L-threonine deaminase	

Note: Manual curator notices an implication of L-serine deaminase and L-threonine deaminse interaction with L-serine and L-threonine. Module 2 only detects an explicit mention of interaction, thus it fails to detect this subtle implication.

Additional Text and Table 4.2- Example result of metabolic interaction from PMID: 13727223

Sentence selected by a manual curator

S1: Dihydroxy acid dehydrase: an enzyme involved in the biosynthesis of isoleucine and valine.

Result achieved from Module 2 within integrated TM framework

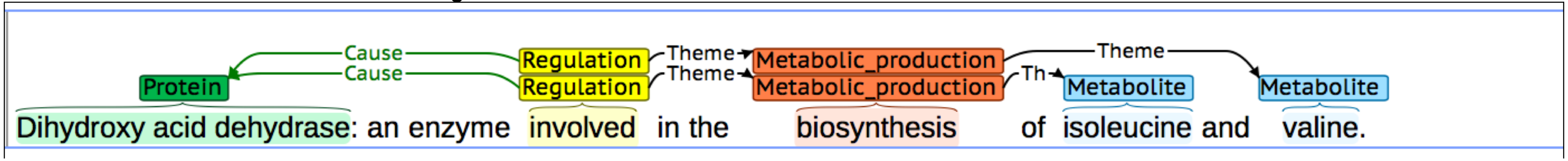

Manual curation	Module 2	
Not found	Dihydroxy acid dehydrase	isoleucine
	Dihydroxy acid dehydrase	valine

Note: This sentence suggests an interaction between Dihydroxy acid dehydrase with isoleucine and valine. However, it can be interpreted either a direct interaction or an indirect interaction. Manual curator can deduce the correct result using domain knowledge. Module 2 detects this sentence as a direct interaction.
