# Supplementary material for: An integrated text mining framework for metabolic interaction network reconstruction
Source: PeerJ. 2016 Mar 21;4:e1811. doi: 10.7717/peerj.1811 (PMC4806637; doi:10.7717/peerj.1811)
Supplement: File S5 [file peerj-04-1811-s005.pdf]

ADDITIONAL FILE 5: The Superpathway of leucine, valine, and isoleucine biosynthesis from metabolic interaction network reconstruction from MEE and MINR modules.

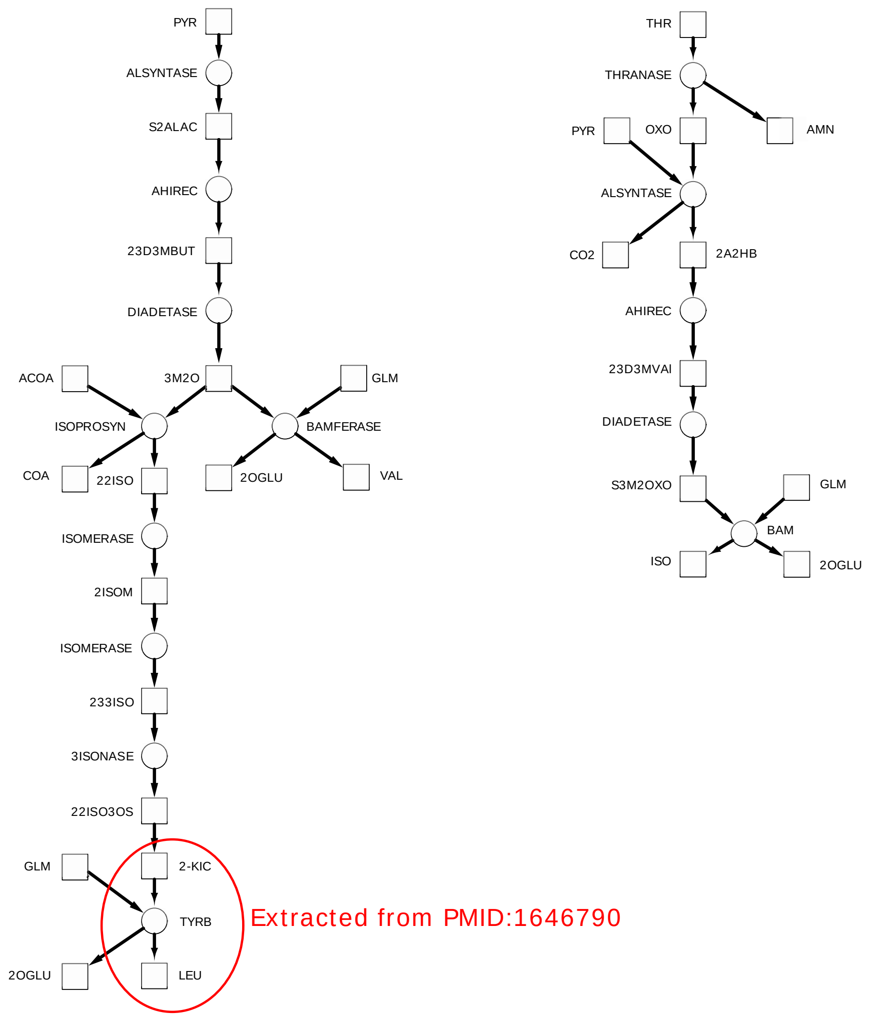

Additional Figure 5.1 – The reconstructed Superpathway of leucine, valine and isoleucine biosynthesis.

Note: This Superpathway is taken from EcoCyc database and is used Cytoscape version 3.0.1. for visualization. A square node means metabolite. A circle node means enzyme. Arrow shows a direction of enzyme-metabolite interaction

Additional Table 5.1 – Full name and abbreviation of metabolites

Full name	Abbreviation
Pyruvate	PYR
(2R,3S)-3-Isopropylmalate	233ISO
(s)-2-Acetolactate	S2ALAC
(2S)-2-Isopropyl-3-oxosuccinate	22ISO3OS
4-Methyl-2-oxopentanoate	2-KIC
2,3-Dihydroxy-3-methylbutanoate	23D3MBUT
L-Leucine	LEU
Acetyl-CoA	ACOA
L-threonine	THR
3-methyl-2-oxobutanoate	3M2O
L-glutamate	GLM
2-Oxobutanoate	OXO
CO_2	CO2
Coenzyme A	COA
2-Aceto-2-hydroxybutanoate	2A2HB
(2S)-2-Isopropylmalate	22ISO
2,3-Dihydroxy-3-methylvalerate	23D3MVAL
2-Oxoglutarate	2OGLU
(S)-3-Methyl-2-oxopentanoate	S3M2OXO
L-Valine	VAL
L-Isoleucine	ISO
2-Isopropylmaleate	2ISOM
Ammonia	AMN

Additional Table 5.2 – Full Name and abbreviation of enzymes

Full name	Abbreviation
Acetolactate synthase	ALSYNTASE
3-isopropylmalate dehydrogenase	3ISONASE
acetohydroxy acid isomeroreductase	AHIREC
tyrosine aminotransferase/branched-chain	TYRB
dihydroxy acid dehydratase	DIADETASE
threonine deaminase	THRANASE
2-isopropylmalate synthase	ISOPROSYN
branched-chain amino-acid aminotransferase	BAMFERASE
isopropylmalate isomerase	ISOMERASE
branched-chain amino-acid	BAM
